# Failure of Oxysterols Such as Lanosterol to Restore Lens Clarity from Cataracts

**DOI:** 10.1038/s41598-019-44676-4

**Published:** 2019-06-11

**Authors:** Damian M. Daszynski, Puttur Santhoshkumar, Ashutosh S. Phadte, K. Krishna Sharma, Haizhen A. Zhong, Marjorie F. Lou, Peter F. Kador

**Affiliations:** 1 Department of Pharmaceutical Sciences, College of Pharmacy, University of Nebraska Medical Center, Omaha, NE USA; 2 Department of Ophthalmology, University of Missouri–Columbia School of Medicine, Columbia, MO USA; 3 Department of Biochemistry, University of Missouri–Columbia School of Medicine, Columbia, MO USA; 4 Department of Chemistry, University of Nebraska at Omaha, Omaha, NE USA; 5 School of Veterinary Medicine and Biomedical Sciences, University of Nebraska at Lincoln, Lincoln, NE USA; 6 Department of Ophthalmology, School of Medicine, University of Nebraska Medical Center, Omaha, NE USA

**Keywords:** Lens diseases, Mechanisms of disease

## Abstract

The paradigm that cataracts are irreversible and that vision from cataracts can only be restored through surgery has recently been challenged by reports that oxysterols such as lanosterol and 25-hydroxycholesterol can restore vision by binding to αB-crystallin chaperone protein to dissolve or disaggregate lenticular opacities. To confirm this premise, *in vitro* rat lens studies along with human lens protein solubilization studies were conducted. Cataracts were induced in viable rat lenses cultured for 48 hours in TC-199 bicarbonate media through physical trauma, 10 mM ouabain as Na+/K+ ATPase ion transport inhibitor, or 1 mM of an experimental compound that induces water influx into the lens. Subsequent 48-hour incubation with 15 mM of lanosterol liposomes failed to either reverse these lens opacities or prevent the further progression of cataracts to the nuclear stage. Similarly, 3-day incubation of 47-year old human lenses in media containing 0.20 mM lanosterol or 60-year-old human lenses in 0.25 and 0.50 mM 25-hydroxycholesterol failed to increase the levels of soluble lens proteins or decrease the levels of insoluble lens proteins. These binding studies were followed up with *in silico* binding studies of lanosterol, 25-hydroxycholesterol, and ATP as a control to two wild type (**2WJ7** and **2KLR**) and one R120G mutant (**2Y1Z**) αB-crystallins using standard MOE^TM^ (Molecular Operating Environment) and Schrödinger’s Maestro software. Results confirmed that compared to ATP, both oxysterols failed to reach the acceptable threshold binding scores for good predictive binding to the αB-crystallins. In summary, all three studies failed to provide evidence that lanosterol or 25-hydroxycholesterol have either anti-cataractogenic activity or bind aggregated lens protein to dissolve cataracts.

## Introduction

The ocular lens is a transparent organ whose function is to focus light onto the retina. It is composed of epithelial cells that are enclosed in a thick capsule formed from epithelial basement membrane^1^. At its anterior surface, the lens contains a single layer of proliferating epithelial cells. As these cells reach the equator, they elongate and differentiate into fiber cells that then make up the bulk of the lens. These elongated fiber cells become completely internalized with their ends joined by collagen at sutures that run from the lens center to anterior and posterior poles. The lens grows throughout life with new fiber cells continually laid on top of the older fiber cells so that the fiber cell depth within the lens is directly related to the age and stage of lens development^2–4^. The transparent lens is unique because this “enclosed bag of regularly ordered cells and proteins” has evolved into an internal micro-circulatory system composed of ions that are coupled to fluid movement that causes the lens to demonstrate behavior similar to that of a single cell^5–7^.

The lens is transparent because light scattering within the lens is minimized. It lacks blood vessels that can scatter and absorb light as well as light scattering cellular organelles such as nuclei, mitochondria, and endoplasmic reticula that are removed during differentiation of the epithelial into fiber cells. Light scattering is further minimized by the specialized organization and composition of the tightly packed fiber cells which contain structural crystallin proteins that also assist in maintaining the proper refractive index in the lens^8,9^. Since the lens fiber cells lack the capacity for protein turnover and repair, specific antioxidant defenses and protein chaperones are present within these fiber cells to protect lens proteins from post-translational changes and aggregation^8,10–13^. Among these are the small heat shock proteins (sHSP) α-crystallins with chaperone-like activity that play a central role in maintaining lens transparency by trapping the denaturing or unfolding proteins that are responsible for light scattering in the highly ordered lens fibers^14–17^.

Cataracts develop from the loss of lens transparency associated with increased light scattering and changes in refractive properties. The protective lens antioxidant defenses and the molecular chaperone reserve of α-crystallin decrease with age so that the aging lens can no longer adequately protect itself from post-translational modifications of lens proteins. This leads to increased light scatter as a function of protein aggregation of post-translationally modified structural proteins which has been experimentally and clinically established in pre-cataractous lenses by dynamic light scattering^14,18,19^. Since this aggregation and denaturation of lens proteins appears irreversible, the surgical removal of the opaque lens is currently the only treatment for restoring vision loss from cataracts^13^. As a result, the development of anti-cataract agents has primarily focused on supplementing the lens with biochemical intermediates or redox agents to reduce or prevent the post-translational modifications that eventually result in irreversible changes in lens protein structure and aggregation. An exception is the pharmacological prevention of diabetic cataracts where a specific enzyme that initiates sugar cataracts has been identified^20–22^.

This paradigm that vision can only be restored through cataract surgery has recently been challenged by Zhao *et al*.^23^ and Makley *et al*.^24^ who report that interaction of lanosterol or 25-hydroxycholesterol with α-crystallin chaperones enhance the ability of these chaperones to restore lens clarity by increasing their ability to physically dissolve protein aggregates and/or the denatured amyloid-like fibril proteins present in cataractous lenses. These reports have subsequently been expanded to include dissolving aggregated proteins in cataracts from two additional congenital mouse models^25,26^. The striking possibility of a non-surgical cataract removal has received worldwide coverage by the news media and encouraged investigators to focus on developing anti-cataract drugs that reverse rather than prevent cataract formation. It has also led to the commercialization of lanosterol eye drops^27–29^. However, the ability of these compounds to restore lens clarity has not been independently confirmed. For example, a recent report has shown that culturing 25 mM lanosterol with the lens nuclei from 40 age-related cataractous human lenses for 6 days at room temperature failed to either dissolve the aggregated proteins or restore the clarity of the lens nuclei^30^. Similarly, clinically administering eye drops containing 5 mM lanosterol dissolved in olive oil two times daily for the first week and three times daily for the next seven weeks to a patient with idiopathic unilateral juvenile nuclear cataracts failed to produce any relevant clinical effect in reversing either the cataract or halting the progressive worsening of visual acuity with an increasing of myopic shift^31^. Triparanol, an inhibitor of the conversion of lanosterol to cholesterol, has also been shown to not only induce cataract formation but also increase tissue lanosterol levels^32–34^. The presence of 25-hydroxycholesterol in human lenses has also been linked to the presence rather than absence of cataracts^35^. To clarify these contrasting findings, we pursued a series of studies to evaluate whether oxysterols such as lanosterol or 25-hydroxycholesterol can reverse experimentally induced cataracts or to re-dissolve aggregated lens crystallin proteins. We also explore if either oxysterols adequately bind to αB-crystallin chaperones at the molecular level.

## Experimental

All procedures involving live animals were performed in accordance with the National Institutes of Health Guide for the Care and Use of Laboratory Animals and the Association for Research in Vision and Ophthalmology Statement for the Use of Animals in Ophthalmic and Vision Research and approved by the Institutional Animal Care and Use Committee (IACUC) of the University of Nebraska Medical Center. Human lenses were obtained from donor eyes from the Saving Sight Eye Bank, Kansas City, MO and stored at −85 °C prior to use.

### *In vitro* lenses culture studies

Eyes from young (125 g, 5-week old) Sprague Dawley rats were immediately enucleated upon death from carbon dioxide asphyxiation. The intact lens from each eye was removed by careful dissection from a posterior approach and cultured as previously described in sterile TC-199 bicarbonate media containing 30 mM fructose and 20 U/mL of penicillin-streptomycin in a humidified incubator under an atmosphere of 95% air and 5% CO_2_ at 37 °C^36–38^.

Following overnight pre-incubation to ensure intact lenses were not damaged during dissection, all clear lenses were transferred into 24-well culture plates containing 2 ml of the standard culture media for 48 hours according to the following 4 groups (6 lenses/group). The first group served as the untreated control, while each lens in the second group was squeezed at the equator with forceps to induce blunt trauma opacities. In the third group, opacities were induced by addition of 10 mM of the  Na/K ATPase inhibitor, ouabain, and in the fourth group opacities were induced by culture with 1 mM of an experimental toxic glycoprotein chaperone that induces osmotic cataract. After 48 hours the culture media for each lens was replaced with fresh TC-199 bicarbonate media containing 15 mM of lanosterol liposomes and the lenses were cultured an additional 48 hours. At the end of the second 48-hour culture period, each lens was carefully washed with PBS solution and transferred to culture plates containing PBS solution. The appearance of each lens was immediately photo-documented by placing the lens over a light source containing a grid. Each photograph was then standardized by adjusting the pixel densities of each outer grid line to a standard value.

### Liposome preparation

Lanosterol (Alpha Chem) was dissolved in a 250 mL round bottom flask containing acetonitrile and the solvent was removed under vacuum in a rotary evaporator. The resulting glassine layer of lanosterol coating the inner surface of the round bottom flask was then removed by scraping with a spatula and then an appropriate amount of TC-199 bicarbonate media was added to the round bottom flask to give a 15 mM solution. The lanosterol sheets were converted to liposomes by sonication using a micro homogenizer tip (MISONIX Fisher Scientific Sonicator Ultrasonic Processor XL) until the solution appeared homogenous (~5 min). Equal aliquots (2 mL) were pipetted into the 24-well culture plates for experimental use.

### Solubilization of proteins from human lens fragments with lanosterol

Two human lenses from a 47-year-old donor were cut through the center of the lens into four equal quadrants and weighed. One piece from each lens tissue was placed in PBS media composed of 137 mM NaCl, 2.7 mM KCl, 10 mM Na_2_HPO_4_, and 1.5 mM KH_2_PO_4,_ pH 7.4, containing 0.03% sodium azide. Lanosterol dissolved in 12.5 µL of ethanol was then added to some of the lens pieces in PBS to give a 0.20 mM lanosterol test solution in a total volume of 500 µL. All lens pieces (with or without lanosterol) were incubated at 37 °C in the dark for 72 hours and then homogenized. The soluble and insoluble protein fractions were separated by centrifugation at 13,000 rpm for 20 min at room temperature in an Eppendorf centrifuge. The protein content of the soluble protein in the supernatant was determined using the Bio-Rad Protein Assay (Hercules, CA). Control samples were similarly prepared using only 12.5 µL ethanol.

### Solubilization of proteins from human lens fragments with 25-hydroxycholesterol

Six frozen lenses from 60-year-old donors were each cut into three equal pie shapes with each sample composed of two pie shapes from different lenses. These formed 9 samples were each immersed into 500 µL of PBS containing 0.03% sodium azide and 10% ethanol containing either 0.0, 0.25 or 0.50 mM of 25-hydroxycholesterol. The samples were then incubated at 37 °C for 72 hours in the dark. Following incubation, the samples were homogenized and the water-soluble fraction was separated from the insoluble fractions by centrifugation at 13,000 rpm for 20 min at room temperature. The water-insoluble fractions were treated with 100 µL of 0.1 M NaOH for 2 hours to solubilize the proteins. The protein content in all samples was measured using the Bio-Rad Protein Assay. Samples incubated only with 10% ethanol served as controls.

### Solubilization of proteins from human lens homogenates with lanosterol

Attempts to solubilize lens proteins by lanosterol and 25-hydroxycholesterol were also carried out using lens homogenates prepared from 72 year old human lenses. Tissue homogenates containing 0.25 and 1.0 mg protein in 0.5 ml PBS (containing protease inhibitor cocktail) and oxysterols at a final concentration of 0, 100 or 200 µM were incubated at 37 °C for 24 hours. At the end of the incubation, the tubes were centrifuged at 13,000 rpm for 15 min at 4 °C. The protein content in soluble and insoluble fractions was measured using Bio-Rad protein assay reagent as described previously. These experiments were conducted three times separately using homogenates prepared from a pair of lenses.

### Molecular modeling studies

**Figure 1:**
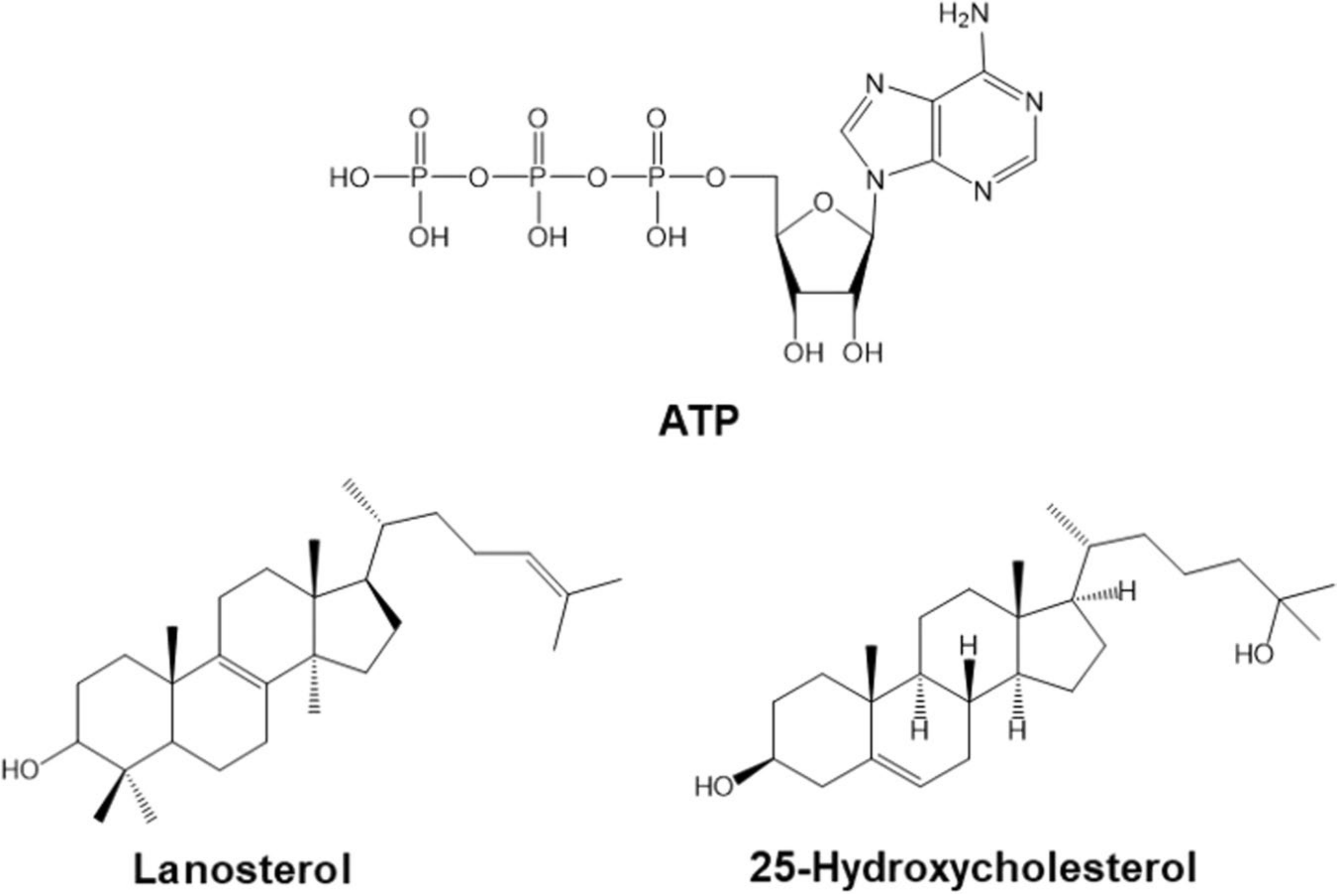
Structures of molecules used in the docking studies with the human αB-crystallin wildtypes, **2WJ7** and **2KLR**, and the R120G mutant, **2Y1Z**.

The Chemical Computing Group’s Molecular Operating Environment 2016 (MOE) and Schrödinger LLC’s program, Maestro 11, were used for this study. The structures (Fig. 1) of ATP, 25-hydroxycholesterol, and lanosterol were built and minimized in MOE^39^. To investigate the binding activities of these three compounds to αB-crystallins, docking studies were conducted using MOE’s internal dock and Schrödinger’s Glide dock^40^. The target proteins were three αB-crystallins obtained from the Protein Data Bank (PDB): a human wild type dimer **2WJ7**^41^, a human wild type αB-crystallin **2KLR**^42^, and a R120G mutant, **2Y1Z**^43^.

All three proteins, wildtype **2WJ7**, wildtype **2KLR**, and R120G mutant **2Y1Z**, were prepared, corrected, and protonated using the Protonate3D function in MOE, followed by energy minimization using Amber14:EHT forcefield with a 0.05 gradient to reduce the steric repulsion. The same proteins were then imported into Maestro and re-prepared using the Protein Preparation Wizard to maximize the hydrogen bond interactions, followed by minimizations with first backbone-constrained and then unconstrained minimization with the OPLS3e forcefield.

### Docking studies

Two binding pockets were defined for the docking studies. The first binding pocket was at the dimer interface as defined by Makley *et al*.^24^ on wildtype **2WJ7**. **2WJ7** is an apo protein without any ligand binding information, and Makley *et al*. did not specify ligand binding residues. We identified PHE 55 on the “A” chain of **2WJ7** as the centroid for the binding pocket extending 25 Å on each side. We defined the same binding pocket for the **2KLR** wildtype, and PHE 118 was identified as the centroid for the R120G mutant dimer, **2Y1Z**. The second binding pocket was the binding site for ATP that was experimentally determined and defined by Ghosh *et al*.^44^ in the β4-β8 groove. This binding mode has also been found in several ATP-binding proteins, including HSP90 and HSP104^45,46^.

Once the binding pocket was identified, ligands were docked to the binding site using MOE docking with the “Triangle Matcher” placement and the flexible “Induced Fit” method. Output was a London ΔG binding score. Predicted K_d_ values were calculated based on the following equation: K_d_ (unit µM) = e^(docking_score*1000/(1.98*298.15))^/10^−6^.

The Maestro docking studies were conducted using Glide Dock by first defining the binding pocket using the Glide Grid Generation method using centroids identified above for all three proteins, wildtypes **2WJ7** and **2KLR** and the R120G mutant **2Y1Z**, followed by Glide dock with scoring function of Extra Precision (XP). The docking scores of each protein-ligand docked complex were expressed in kcal/mol, with −9 kcal/mol approximated to an IC_50_ or K_d_ in the high nM/low μM range. A more negative docking score indicates stronger ligand binding to the protein.

## Results

### Organ culture studies

**Figure 2:**
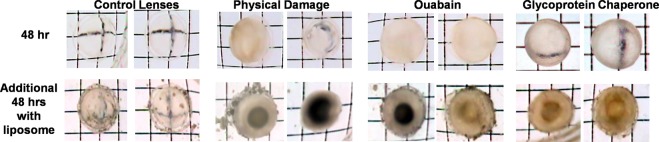
Appearance of rat lenses after 48-hour incubation following the induction of cataract and subsequent additional incubation with 15 mM of lanosterol liposomes. While the control lenses appear relatively clear after 48-hour exposure to lanosterol, the presence of lanosterol did not halt the further progression of cataracts. Lens photos were taken over a grid with special care to standardize the outer grid size and intensity in comparison to the control lenses since changes in intensity can affect the apparent levels of lens opacity.

The new paradigm suggests that lanosterol and other oxysterols are able to clear cataracts, presumably by boosting the α-crystallin’s chaperone activity to dissolve the light scattering, denatured, aggregated lens proteins responsible for opacification. Since transparency and biochemical viability can be maintained for up to two weeks, lens organ culture has become a valuable technique in investigating the mechanisms of both lens homeostasis and cataractogenesis^47,48^. Therefore, lens culture studies were conducted to investigate the ability of lanosterol to reduce lens opacities. Cataracts were experimentally induced with blunt trauma, by ATPase ion transport inhibition with 10 mM of ouabain, or by osmotic stress induced with 1 mM of a toxic experimental compound that induces water influx into the lens. Untreated lenses were used as the control. Within 48 hours of culture, all the lenses developed opacities except the control untreated group. As illustrated in Fig. 2, trauma-induced cataracts appeared as localized opacities that varied because of the levels of blunt physical trauma applied, while the ouabain-treated lenses developed cortical opacities and the osmotic inducer produced dramatic refractive index changes associated with significant lens swelling. All lenses were then transferred to similar TC-199 bicarbonate media containing 15 mM of lanosterol liposomes. Incubation for an additional 48 hours in the lanosterol liposome media resulted in an apparent penetration of the amber liposomes into the lenses as noted by the slight color changes, especially around the nucleus as all treated lenses progressed to the mature nuclear cataract stage. Over the 48-hour period, there was also liposome clumping at the surface of the collagen capsule which was difficult to rinse off, presumably because of the sticky nature of the liposomes. While transparency in the control lenses appeared to be minimally affected, lanosterol failed to reverse opacities or halt the progression of cataracts in all treated lenses. In fact, lens opacities in all treated lenses exposed to lanosterol progressed to the more advanced mature cataract stage with apparent nuclear involvement.

### Protein binding studies

**Figure 3:**
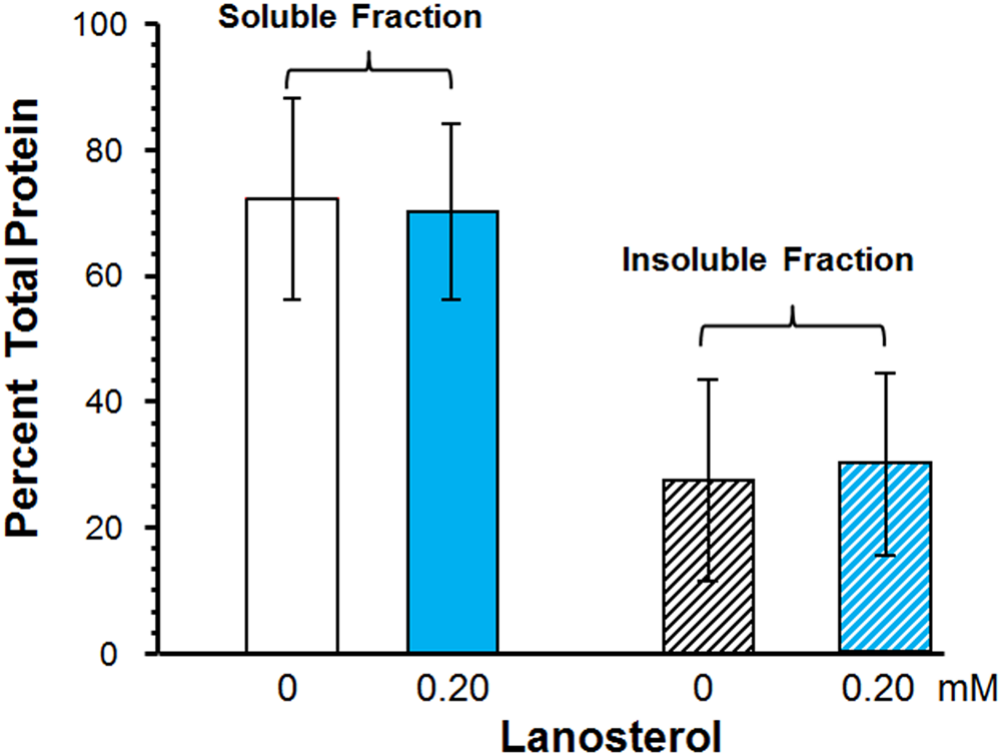
Change in protein levels from 47-year old human lens fragments after 3 days incubation at 37 °C in media containing 0.20 mM lanosterol. There is no change in the levels of proteins in the soluble and insoluble fractions with or without the presence of lanosterol. p > 0.5 ANOVA single factor analysis and n = 4 ± S.D.

The above described lens culture studies suggest that lanosterol either inadequately binds to lens proteins or that the chaperone activity of the assumed bound lanosterol to α-crystallin complex is not adequate to reverse or alter the progression of cataracts as previously proposed. To evaluate this possibility, two lenses from a 47-year-old human were equally divided into 4 pie shaped portions and incubated in the dark for 3 days at 37 °C in media containing 0.2 mM lanosterol dissolved in 10% ethanol. As shown in Fig. 3, incubation with lanosterol did not result in either an anticipated increase in the level of soluble lens proteins or decrease the levels of insoluble proteins. This indicates that lanosterol failed to solubilize the insoluble lens proteins present in the cataractous lens. Similar results were obtained with three lenses from 70-year-old humans analyzed separately (data not shown).

**Figure 4:**
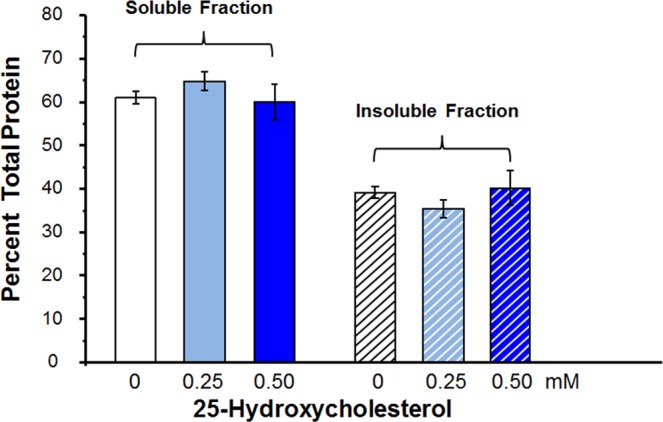
Change in soluble/insoluble protein levels of 60-year-old human lens homogenate after 3 days incubation at 37 °C in media containing either 0.0, 0.25 or 0.50 mM of 25-hydroxycholesterol. There is no change in the levels of proteins in the soluble and insoluble fraction. n = 3 ± S.D. p = 0.79 by ANOVA single factor analysis.

Since in our experiments lanosterol failed to increase soluble protein levels, the binding studies were expanded to include 25-hydroxycholesterol whose binding power has been reported to be superior to that of lanosterol^24^. For these studies, 6 frozen lenses from three 60-year-old donors were each cut into three equal pie shaped fragments, thawed and incubated in the dark for 3 days at 37 °C in media containing either 0.0, 0.25 or 0.50 mM of 25-hydroxycholesterol. For each group, two pieces from different lenses were combined for each experiment. The percentage of protein in soluble and insoluble fractions were estimated separately for all samples in each group and averaged. As summarized in Fig. 4, no significant difference in soluble protein levels (p-value of 0.79, by ANOVA single factor analysis) was observed between the non-treated and 25-hydroxycholesterol-treated lenses. If the aggregated proteins were solubilized, then the levels of proteins in the insoluble fractions should have been lowered in the 25-hydroxycholesterol treated lenses. Instead, the insoluble protein levels in the control and the groups treated with either 0.25 or 0.50 mM 25-hydroxycholesterol showed no difference.

**Figure 5:**
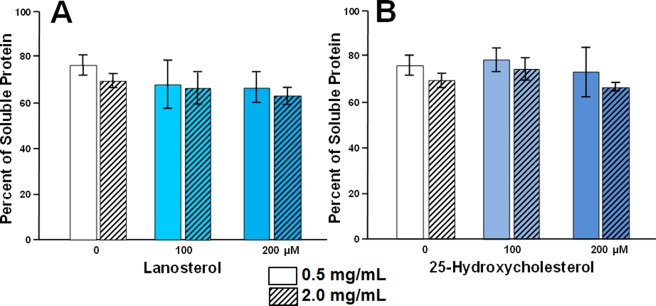
The solubility of human lens proteins in the presence of (**A**) lanosterol and (**B**) 25-hydroxycholesterol. Tissue homogenates from 72 year old human lenses containing 0.5 or 2.0 mg protein in 1.0 mL of PBS were incubated with 0.0, 100 and 200 µM lanosterol or 25-hydroxycholesterol at 37 °C for 24 hours. The values shown are the percentage of protein remaining in the soluble fraction after incubation. N = 3 ± S.D. and p > 0.05 ANOVA single factor analysis.

The lack of binding demonstrated by both oxysterols could possibly be due to the failure of both oxysterols to adequately penetrate the lens fibers in the incubated lens fragments. To investigate this possibility, protein solubilization studies with 100 and 200 µM concentrations of lanosterol and 25-hydroxycholesterol were carried out with lens homogenates of 0.5 and 2.0 mg/mL protein concentrations in PBS (containing protease inhibitor cocktail) prepared from 72 year old human lenses. Changes in the soluble protein levels after 24-hour incubation at 37 °C are illustrated in Fig. 5. The results confirm that lanosterol and 25-hydroxycholesterol do not affect lens protein solubilization when lens homogenate was used.

### Molecular modeling studies

Since both oxysterols failed to increase the anticipated soluble protein levels in lenses or lens homogenates where age-related protein denaturation had occurred, this suggests that oxysterol binding to the crystallin chaperones may be inadequate. Therefore, additional *in silico* studies to gain insight into chaperone binding to lanosterol and 25-hydroxycholesterol at the molecular level were conducted. As specified in the Method Section, lanosterol and 25-hydroxycholesterol were docked to three small αB-crystallins heat shock chaperones, two human wild type dimers, **2WJ7**^41^ and **2KLR**^43^, and an ARG120GLY mutant, **2Y1Z**^43^. As an additional control, ATP, which has been reported to bind these αB-crystallins, was also examined. Molecular modeling and docking studies were conducted using MOE dock and Schrödinger Glide dock standard docking methods. As specified in the Method section, the appropriate binding modes were determined by docking the ligands to two potential pockets, an ATP pocket and the dimer interface. The top five docked poses were collected and the best docking scores along with predicted K_d_ values are presented. The MOE dock scores are reported as London ΔG binding scores. London ΔG binding scores generally indicate that a value of −12.00 corresponds to the low nM range and a dissociation constant, K_d_, of −12.3 kcal/mol, a value of −8.00 corresponds to the low µM range and a K_d_ of −8.2 kcal/mol, and a value of −4.00 corresponds to the low mM range and a K_d_ of −4.1 kcal/mol.

The MOE docking results, summarized in Table 1, show that good ATP binding to the ATP binding pocket in the wild type **2WJ7** and **2KLR** (~−2 kcal/mol) were observed. This indicates that the methods employed successfully predicted the expected feasible nanomolar range binding of ATP to the ATP binding pocket of these chaperones. However, ATP binding to mutant **2Y1Z** appeared to be lower as suggested by the lower docking score (−9.82 kcal/mol). In contrast, the MOE docking results suggested only weak binding of lanosterol (−4.33 kcal/mol) and 25-hydroxycholesterol (−6.75 kcal/mol) to the dimer interface containing the PHE centroid in the wild-type **2WJ7** model. Furthermore, the results predict that no binding of either oxysterols to **2KLR** and **2Y1Z** is anticipated because positive docking scores suggest unfavorable binding. A value of “n.d.” indicates that no docking data could be acquired.

**Table 1:** MOE docking scores of select compounds (Fig. 1) to their respective binding pocket (i.e., ATP to the ATP binding pocket and the sterols to the dimer interface binding site) of the wild-types, **2WJ7** and **2KLR**, and the R120G mutant, **2Y1Z**. A value of “n.d.” indicates that no docking data could be acquired after 30 iterations.

Compounds	2WJ7 Wildtype		2KLR Wildtype		2Y1Z Mutant	
	Best	Pred K_d_	Best	Pred K_d_	Best	Pred K_d_
ATP	−12.72	0.44 nM	−11.48	3.59 nM	−9.82	59.6 nM
25-Hydroxy-Cholesterol	−6.75	10.82 μM	0.11	n.d.	11.33	n.d.
Lanosterol	−4.33	652.44 μM	2.34	n.d.	17.80	n.d.

Could the oxysterol binding perhaps occur at the ATP binding site? To address this question, docking studies with ATP, 25-hydroxycholesterol and lanosterol were also conducted using the Glide dock program to the β4-β8 groove, which is the ATP interactive binding region, as well as the dimer interface containing the PHE 55 centroid (Table 2). Again, adequate binding values for ATP binding to the ATP binding pocket of the wild-type **2WJ7** and **2Y1Z** mutant models were obtained; however, oxysterols were not able to bind to the ATP binding pockets of these αB-crystallins. Adequate ATP binding onto the dimer interface of the **2WJ7** model was also observed, however, the binding of ATP to the interface of the mutant **2Y1Z** was much weaker, suggesting that ATP would preferentially bind to the ATP pocket rather than the dimer interface. This is not surprising since natural selection has preferred ATP to bind to the ATP binding pocket. Only very weak oxysterol binding to the dimer interface of the αB-crystallins was observed, suggesting that the oxysterols are only very weak binders or do not bind at all to either the ATP binding pocket or at the dimer interface.

**Table 2:** Maestro docking scores and predicted K_d_ values of ATP and oxysterols to wildtypes **2WJ7** and **2KLR** and mutant **2Y1Z**. All compounds were docked to the ATP interactive binding region containing the Walker-B motif, as well as the dimer interface with PHE 55 on the “A” chain defined as the centroid. A value of “n.d.” indicates that no docking data could be acquired at 30 iterations. The predicted K_d_ values are reported in µM concentrations.

Compounds	β4-β8 Groove, ATP Interactive Binding Region						Dimer Interface with PHE 55 Centroid					
	2WJ7 Wildtype		2KLR Wildtype		2Y1Z Mutant		2WJ7 Wildtype		2KLR Wildtype		2Y1Z Mutant	
	Best	Pred K_d_	Best	Pred K_d_	Best	Pred K_d_	Best	Pred K_d_	Best	Pred K_d_	Best	Pred K_d_
ATP	−8.23	0.88	−5.57	79.86	−9.65	0.08	−9.43	0.12	−4.31	674.92	−5.67	67.41
25-Hydroxy-Cholesterol	−3.20	4424.35	n.d.	n.d.	n.d.	n.d.	−4.40	579.49	−3.96	1221.06	−2.87	7737.86
Lanosterol	n.d.	n.d.	n.d.	n.d.	n.d.	n.d.	−3.33	3549.86	−1.54	73631.71	n.d.	n.d.

Therefore, as summarized in Tables 1 and 2, binding studies conducted using MOE dock and Schrödinger Glide dock methods support the protein binding studies that indicate that lanosterol or 25-hydroxycholesterol do not appear to adequately bind to αB-crystallins.

## Discussion

The mammalian lens contains millions of densely packed fiber cells that are continuously formed throughout life from differentiating lens epithelial cells. These fiber cells contain three major crystallin proteins families: α-crystallin, which resembles small heat shock proteins, and β- and γ-crystallins, that have structural and functional roles in maintaining transparency and the high refractive index of the lens. Increased light scattering leading to the appearance of lens opacities is directly linked to lens protein aggregation of the β- and γ-crystallins. To counteract this aggregation, Horwitz^16,17,49^ has proposed that small heat-shock proteins, αA- and αB-crystallins, serve as chaperones that protect the lens against protein aggregation. The protein unfolding hypothesis for age-related cataract postulates that the progressive modifications of β- and γ-crystallins reduce their free energies of unfolding and promote their binding to α-crystallin. By binding to α-crystallins, lanosterol and 25-hydroxycholesterol have been proposed to enhance the ability of α-crystallin to bind to the unfolded (aggregated) β- and γ-crystallins. Solubilizing these insoluble, light scattering aggregated lens proteins should result in an increase of soluble proteins and a decrease in insoluble proteins, *i*.*e*. “the cataract should be dissolved”.

Cholesterol derivatives, in addition to interacting with crystallins, also play an integral role in the regulation of cholesterol-dependent processes in fiber cell plasma membranes and in the maintenance of fiber cell membrane homeostasis^50^. With the loss of its organelles during fiber cell differentiation, the plasma membrane forming the external boundary of the fiber cell cytoplasm becomes the only remaining membrane in mature fiber cells^51^. Its extremely high cholesterol content makes this membrane one of the most saturated and ordered (stiff) membranes in the human body. While the need for this high lipid content is unclear, disturbances of cholesterol homeostasis can lead to cataract formation.

The first synthetic cholesterol lowering drug associated with irreversible cataract formation was triparanol (MER-29)^52^. This compound inhibited cholesterol synthesis at the desmosterol step, several steps downstream of lanosterol and resulted in the cellular accumulation of lanosterol^33,34^. Although lens lanosterol levels were never specifically measured after triparanol administration, it can be assumed that downstream inhibition of cholesterol synthesis would similarly increase lanosterol levels in the lens. Therefore, altered cholesterol homeostasis may lead to cataract formation despite an increase in lanosterol. Similarly, cataracts have been associated with the lenticular accumulation of cholesterol oxides such as 25-hydroxycholesterol^35^. Cataract formation has also developed with other cholesterol lowering agents such as statins and fibrates. Animal studies and several clinical studies report that patients undergo cataract surgery at higher rates with long-term statin or fibrate administration^37,53–56^. Statins inhibit cholesterol biosynthesis at the initial mevalonic acid level, while fibrates modify lipids, decrease triglycerides, and alter cholesterol levels of HDL/LDL by activating alpha peroxisome proliferator-activated receptors (PPAR-α). Studies suggest that cataract formation is induced by statins because they lower the isoprenylation of small GTPases^57^. At present, the relationship between the regulation of cholesterol-dependent processes in lens fiber cells and their plasma membranes and cataract formation is not well established and more studies in this area are required.

*In vitro* organ culture of lenses has become a powerful experimental tool for not only investigating the relationship between lens metabolism and lens clarity, but also for elucidating the mechanism(s) of cataract formation by drugs and biochemical agents^47,58^. These culture studies require carefully excised lenses that are cultured in specially buffered and osmotically compensated culture media at 37 °C in order to maintain their viability and clarity^59,60^. Moreover, because the use of freshly excised lenses are ideally required, the majority of culture studies employ readily available rat lenses. Rat lens organ culture studies have been used to elucidate the effect of statins on cataract formation^37,57^, the role of osmotic changes on sugar cataract formation^38,61^, and the role of oxidative stress on oxidation induced cataracts^62,63^.

In the present organ culture studies, freshly excised clear rat lenses were incubated under established conditions. During the initial 48-hour culture period, cataracts were induced in select groups of these clear lenses with either physical blunt trauma, inhibition of ATP, or experimentally induced osmotic stress. Blunt traumatic injury results in localized lens membrane damage and increased oxidative stress^64,65^, while ouabain alters the intracellular Na+ and K+ levels and affects the anabolism and catabolism of protein during cataract formation^66^. Experimentally induced osmotic changes initiate increased endoplasmic reticulum (ER) stress in lens epithelial cells that subsequently results in the generation of reactive oxygen species (ROS) and oxidative stress^67–69^. These biochemical insults all lead to similar protein destabilization, the presence of partially unfolded aggregation-prone intermediates, and the formation of insoluble, light-scattering protein aggregates that appear as lens opacities^13^. Subsequent 48-hour exposure of these lenses to 15 mM of lanosterol liposomes failed to decrease the insoluble, light-scattering protein aggregates that had developed into lens opacities. More importantly, the presence of lanosterol during this 48-hour period also had no effect on influencing the further progression of protein aggregate development because all lens opacities progressed to the advanced nuclear stage (Fig. 2). Similarly, Shen *et al*. observed that lanosterol failed to prevent opacities induced by U18666A, an agent that induces cataracts in part by inhibiting the formation of lanosterol^70^. This is in contrast to the report by Zhao *et al*.^23^ who report that the clarity of cataractous rabbit lenses was improved in culture. Their culture condition was quite different from ours as we ensure the viability of the cultured lenses by incubating under 37 °C physiological temperature, and we also paid extra attention to ensure that the lens photos (Fig. 2) taken after incubation were standardized to the same contrast and exposure, and by adjusting the pixel densities of each outer grid line to a standard value.

These lens organ culture results, which strongly suggest that there was no apparent interaction between lanosterol and the experimentally aggregated lens proteins, concur with the subsequent protein binding studies (Figs 3–5) where lanosterol or the presumably more potent 25-hydroxycholesterol both failed to interact with aggregated proteins in human lenses to increase the levels of soluble proteins by decreasing the levels of insoluble proteins. These results are in agreement with those of Shanmugam *et al*.^30^ in which the authors also failed to show any change in soluble protein levels in 40 nuclear cataracts cultured with lanosterol. It is well-known that fully denatured proteins lack both tertiary and secondary structure and exist as so-called random coils where the only fixed relationship between the amino acids is the peptide bond between adjacent residues. While Zhao *et al*.^23^ and Makley *et al*.^24^ both focused on the misfolded crystallins only existing as amyloid fibrils characterized by intermolecular cross-β-sheet formations and relatively ordered morphologies^71,72^, crystallin aggregates can also adopt alternative amorphous forms other than amyloid fibrils^73–75^. This may explain, in part, the observed inability of aggregated lens proteins to be re-dissolved in the present studies. A recent study reported that sonicated human lens homogenates were partially solubilized by lanosterol and 25-hydroxycholesterol when the samples were kept in a shaker for 14 days^76^. Since it is known that sonication disaggregates and solubilizes water-insoluble lens proteins^77^, it is unclear whether the prior sonication of the sample and several days of shaking contributed to the ability of sterols to partially solubilize the lens proteins. In another study^78^, treatment of sonicated bovine lens extract with 50–500 µM 25-hydroxycholesterol for two days did not result in significant reduction of turbidity. Further, it is yet to be determined whether the results of lens protein solubilization observed after sonication and shaking with sterols can be interpreted as *in vivo* therapeutic potential of the sterols since both sonication and shaking are not treatment modalities.

Failure of lanosterol in our studies to alter the appearance of formation of cataracts, along with the inability of oxysterols to reduce insoluble protein levels, prompted us to examine if oxysterols can bind to the crystallin chaperones at the molecular level to enhance chaperone function to re-dissolve aggregated proteins. Using both MOE dock and Schrödinger’s Glide dock programs, *in silico* docking studies confirmed that both lanosterol and 25-hydroxycholesterol failed to reach the therapeutically significant low micromolar range binding (docking score, ≤−8) with wild-types **2WJ7**, **2KLR** and mutant **2Y1Z** αB-crystallins chaperones (Tables 1 and 2). To confirm our methodology, the present studies used ATP as a control since ATP is known to bind to αB-crystallin chaperones^46^. While overall the docking scores from the MOE dock program appeared to overestimate binding compared to the Glide dock program, feasible binding with ATP was observed by both programs. Glide dock docking scores (Table 2) for **2KLR** and **2Y1Z** models gave ATP binding scores (−5.57 kcal/mol at the ATP pocket of **2KLR** and the interface of **2KLR** and **2Y1Z**) that were in good agreement with Palmisano *et al*.^79^ who determined the binding constant for ATP to the α-crystallin to be (K_a_ = 8.1 * 10^3^ M^−1^ which corresponds to −5.33 kcal/mol when converted to ΔG). Significantly better ATP binding scores were obtained for binding at the ATP binding pocket of **2WJ7 and 2Y1Z** (−8.23 kcal/mol and −9.65 kcal/mol, respectively), and at the interface with **2WJ7** (−9.43). In contrast to ATP, the present MOE and Glide docking scores suggest that lanosterol and 25-hydroxycholesterol binding to α-crystallin is unlikely at less than micromolar concentrations. For example, the K_d_ for lanosterol and 25-hydroxycholesterol, respectively, are predicted to be 653 µM and 11 µM against wt **2WJ7** (Table 1, MOE dock), and 3.55 mM and 579 µM (Table 2, Glide dock). For 25-hydroxycholesterol, the predicted K_d_ for **2Y1Z** is at 7.74 mM concentration (Table 2). It is unlikely that these high micromolar or even millimolar concentrations can be clinically achieved in the lens.

Our docking results were not contradictory to previously reported data in Makley *et al*. and Zhao *et al*. In Makley *et al*., 100 µM concentration of drugs were used and at this concentration, 56% recovery vs wild-type in T_m_ was observed^24^. In Zhao’s paper, the observation of “reduction in cataract severity” was achieved in using 25 mM concentration of lanosterol^23^. The free energy ΔG for K_d_ of 25 mM range would be −2.48 kcal/mol. A ligand with a K_d_ above 10 µM (dock score: worse than −6.8) is normally considered to be low affinity. Both our docking results, and the fact that the Makley’s and Zhao’s experiments used high concentration (from 100 µM to 15 mM), suggest that under 10 µM or lower concentration, it is unlikely for lanosterol to have significant and specific binding to α-crystallin.

**Figure 6:**
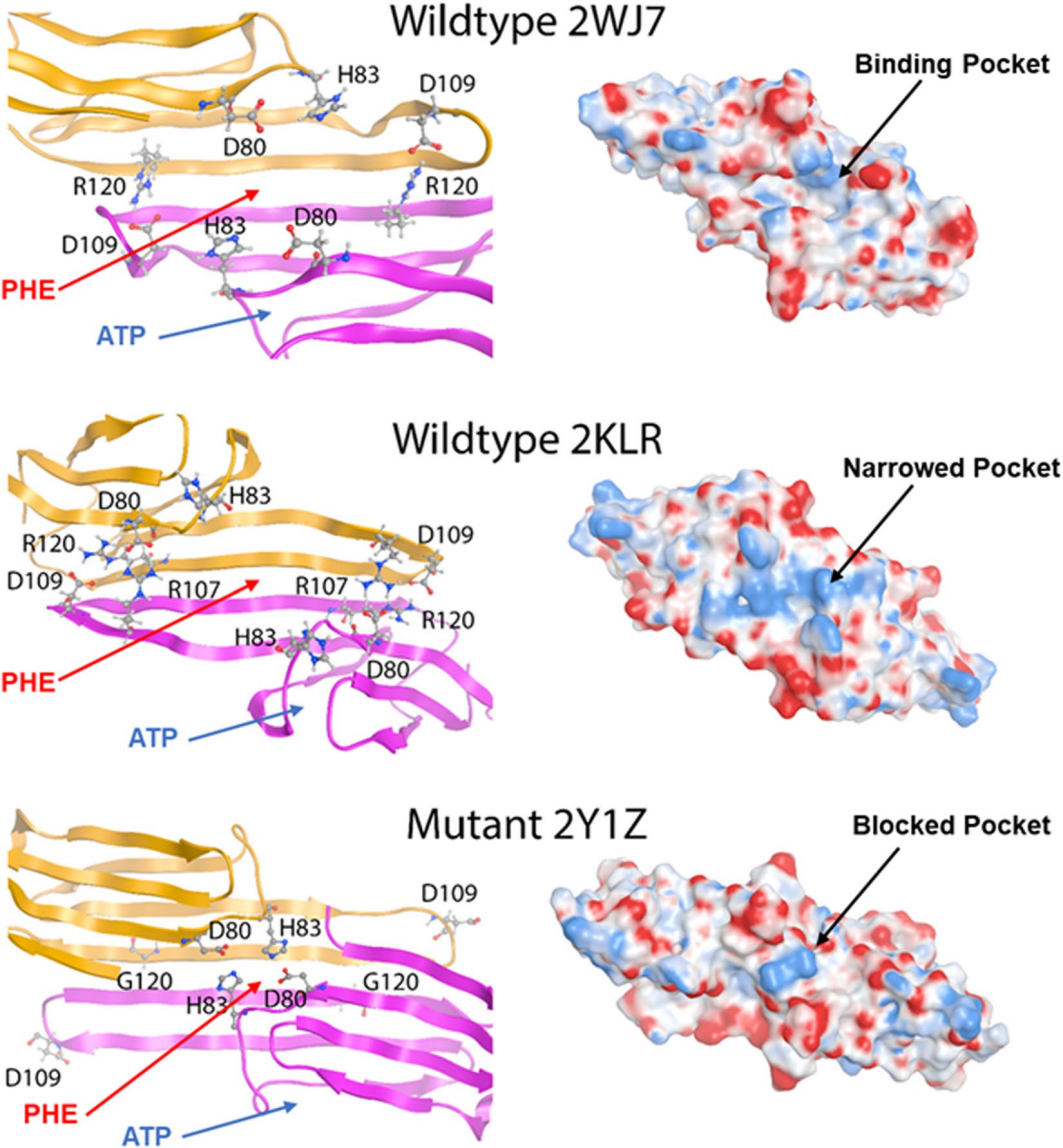
Comparison of the surface and charge differences (sphere structure), along with the location of key amino acid residues (ribbon structure) between the αB-crystallin wildtypes **2WJ7** and **2KLR** and the ARG120GLY mutant **2Y1Z**. Locations of the defined ATP pocket and PHE 55 binding regions for docking studies are marked with arrows on the ribbon structures. The experimentally determined ATP-binding site is in the β4-β8 pocket where the amino acid sequence ^82^KHFSPEELKVKVLGD^96^ resembles the Walker-B ATP-binding motif. PHE 55 is in the centroid of the dimer interface. Note the presence of the open groove at the dimer interface in **2WJ7**, which is nonexistent in **2KLR** and shielded by the ASP 80/HIS 83 salt bridge in **2Y1Z**. These protein models are in agreement with previously reported results^38^.

Additional protein-ligand interaction in our studies further disclosed the underlying cause for the poor binding of oxysterols, such as lanosterol, to the current αB-crystallins models. The human wild-type dimer **2WJ7** contains residues ASP 109 and ARG 120, (Fig. 6) which were identified to be synonymous with those described in Makley *et al*.^24^. These residues are located at the dimer interface where the carboxylic group of ASP 109 on one chain and the guanidine group of ARG 120 on the opposite chain form a salt bridge that contributes to the protein’s stability. This open groove binding pocket is shown to be hydrophobic (white) and surrounded by positively charged residues (blue). Inspection of this dimer interface reveals that the central open groove binding pocket identified in **2WJ7** becomes narrower in **2KLR** and is blocked in **2Y1Z**. **2KLR** has a second salt bridge between ASP 80 and ARG 107 which further stabilizes the dimer interface so that a closed-groove conformation can be maintained. Such a scenario may result in a poor binding environment for both lanosterol and 25-hydroxycholesterol. This structural difference helps to explain why the docking scores of ATP and oxysterols are smaller in **2KLR** than those of **2WJ7**. Similarly, the open-structure observed in wild type **2WJ7** is disrupted in the ARG120GLY mutation **2Y1Z** so that the mutation of ARG120 to GLY makes it impossible to form a stabilizing salt bridge between ARG120 and ASP109. Instead, salt bridges between ASP 80 and HIS 83 are formed in which the side chains of these two residues block the central pocket so that no suitable binding with either oxysterol can occur.

Since no crystal structures of the αB-crystallin/oxysterol complex are available, and the published papers, including that of Makley *et al*.^24^, have not identified specific binding residues used for docking oxysterols to αB-crystallins, residue PHE 55 of the “A” chain was chosen as the centroid to define the binding pocket for the dimer interface in **2WJ7**, **2KLR** and **2Y1Z**. In addition, the Walker-B ATP-binding motifs containing the sequence ^82^KHFSPEELKVKVLGD^96^ were used for docking on both wildtypes, **2WJ7** and **2KLR** and mutant **2Y1Z** to determine whether the oxysterols can also bind to the ATP binding pocket. This ATP binding pocket was previously reported by Ghosh *et al*.^44^. Both binding regions are highlighted in Fig. 6.

**Figure 7:**
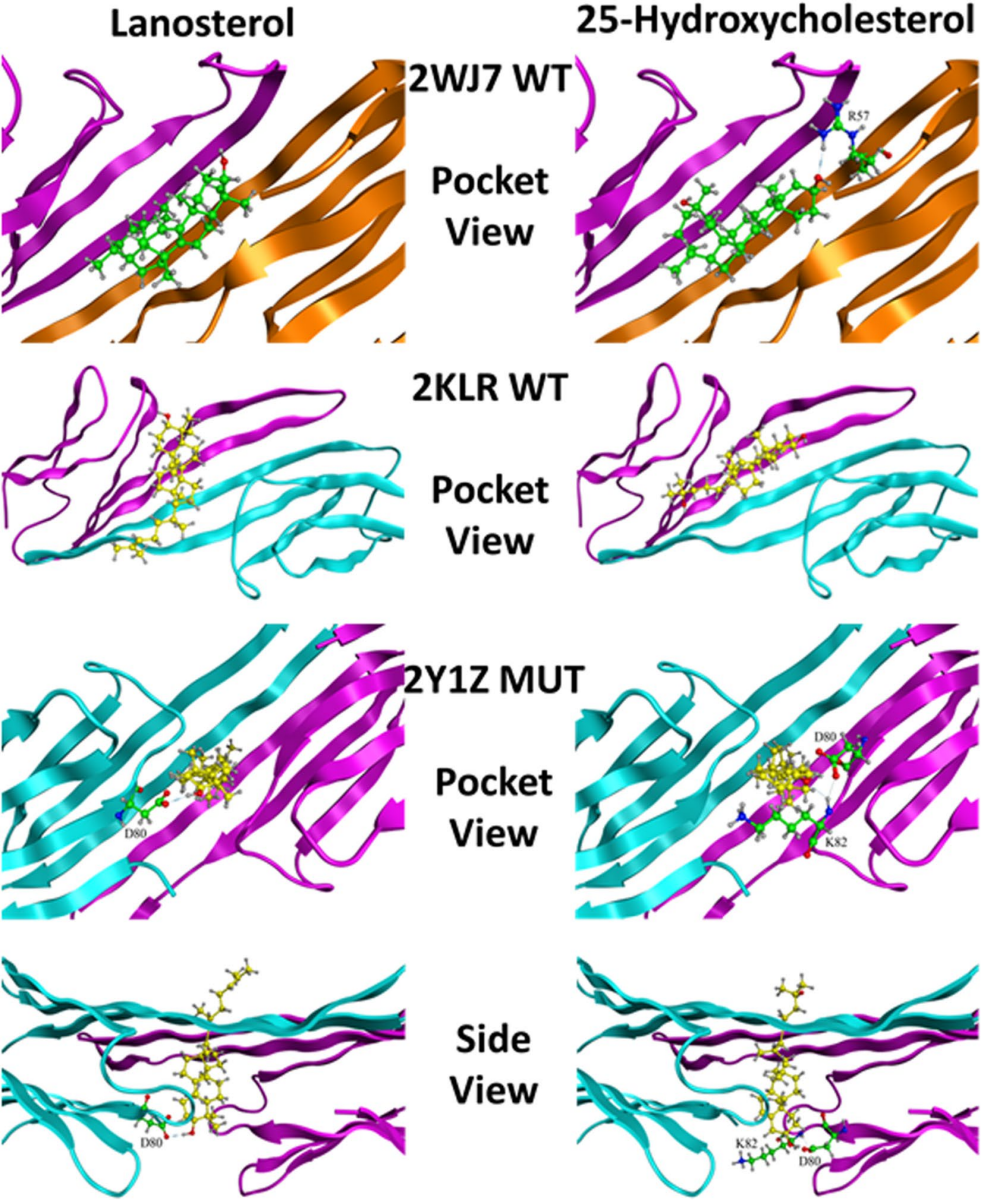
MOE ribbon structures depicting the best docked complexes for lanosterol and 25-hydroxycholesterol with the wildtype and mutant αB-crystallins. Note that neither oxysterol could enter the dimer interface in the 2Y1Z mutant and the only interaction was perpendicular to the interface pocket.

Because an ATP binding site on αB-crystallins has previously been identified, successful binding of ATP to this site was easily and successfully achieved. As discussed above, the Glide docking scores were in better agreement with the experimental data than those from the MOE dock method^79^. Depending on the αB-crystallin model employed, the binding constant for ATP was estimated to be in the range of 0.1 to 80 µM (Table 2). However, achieving similar binding results for the oxysterols was more difficult. No suitable binding pose for lanosterol was identified. For 25-hydroxycholesterol, the predicted K_d_ values were between 4.4 mM and 27.6 mM for the **2WJ7** and **2Y1Z** model, respectively. It is not surprising that the narrowed ATP binding pocket (Fig. 6) is unable to accommodate the large oxysterol molecules. For the larger dimer interface, a docked pose for 25-hydroxycholesterol was identified; however, the predicted K_d_ values for **2WJ7**, **2KLR**, and **2Y1Z**, were 579 µM, 1.22 mM, and 7.74 mM, respectively. Binding results for lanosterol in the dimer interface were even worse, with K_d_ values for **2WJ7** and **2KLR** of 3.55 mM and 73.63 mM obtained; K_d_ values for **2Y1Z** could not be determined. In addition, no suitable docked pose for the **2Y1Z** mutant was identified. Inspection of the protein structures and docked ligands showed that the 3-hydroxyl group of 25-hydroxycholesterol can form a favorable hydrogen-bond with ARG 57, which is missing in the similar binding of lanosterol. This hydrogen-bond was absent in the **2KLR** and **2Y1Z** models (Fig. 7). Therefore, 25-hydroxycholesterol showed even worse binding activity (mM range, Table 2). Furthermore, blocking of the dimer interface in the **2Y1Z** model due to the salt bridge of Asp80 and HIS 83 made it unlikely for either oxysterol can bind (Figs 6 and 7). The only interaction between the oxysterols and the dimer interface was perpendicular to the interface pocket (Fig. 7).

**Figure 8:**
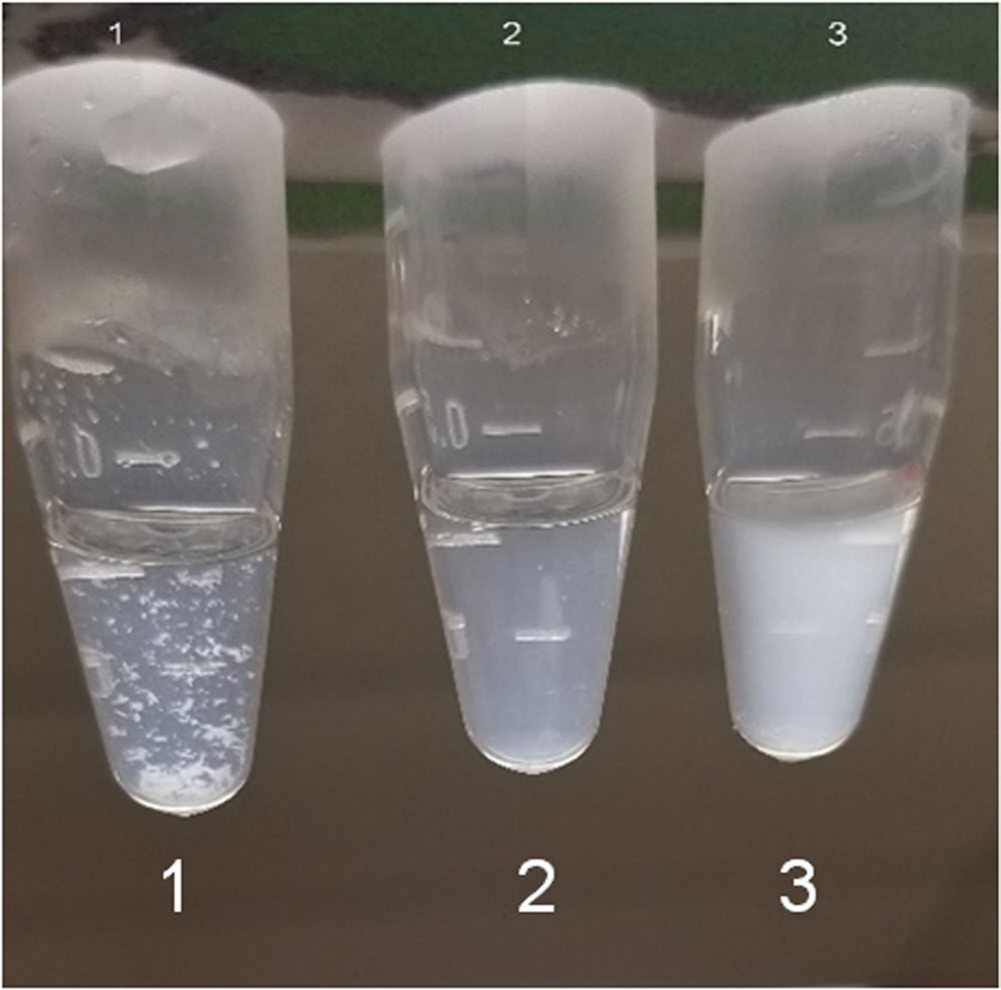
Appearance of 25-hydroxycholesterol dissolved in PBS solution with or without the presence of αB-crystallin. (**1**) 25-hydroxycholesterol (0.25 mM) in PBS. (**2**) αB-crystallin (2.0 mg/mL) in PBS. (**3**) 0.25 mM 25-hydroxycholesterol +2.0 mg/mL αB- crystallin.

Despite the poor binding results of 25-hydroxycholesterol with the three αB-crystallin models and the inability of 25-hydroxycholesterol to solubilize lens proteins (Figs 4 and 5), some interaction of αB-crystallin with this oxysterol can occur. As illustrated in Fig. 8, when 25-hydroxycholesterol is placed in a solution of PBS, it remains as a precipitate and fails to dissolve into the solution. However, αB-crystallin does dissolve in PBS. Combining both PBS solutions together results in the formation of a white turbid solution. While the oxysterol indeed appears to solubilize in the αB-crystallin solution, subsequent multi-angle light scattering (MALS) analysis indicates that the 25-hydroxycholesterol becomes trapped within the oligomers of αB-crystallin. The complex peak showed no significant change in the molar mass (MW) or hydrodynamic radii (R_h_) of αB-crystallin incubated with 25-hydroxycholesterol. Consistent with the present docking and binding studies, this suggests that 25-hydroxycholesterol is held within αB-oligomers without any apparent binding interaction with the protein. This interaction may be similar to the partitioning of dexamethasone with α-crystallin as reported by Augusteyn and co-workers and interpreted as a non-functional interaction^80^.

Lens protein aggregation occurs within the millions of mature lens fiber cells where αB-crystallin chaperones are located. Therefore, the oxysterols must penetrate into the millions of fiber cells to bind to the αB-crystallin chaperones. While investigators have focused on lens protein aggregation and the role of oxysterol induction of chaperones in reversing lens protein aggregation in solutions, missing is the consideration of the unique properties of the lens fiber membranes and the required demonstration that oxysterols can actually penetrate through the highly saturated, stiff cholesterol containing membranes of these fiber cells which are tightly interconnected through ball and socket junctions. In fact, in the rat lenses cultured with lanosterol liposomes (Fig. 2), the amber appearance of the cortex and surrounding nucleus in the cataractous lenses does not rule out the possibility that the appearance is due only to the presence of liposomes in the extracellular space between the lens fibers. However, the failure of both oxysterols to solubilize lens protein homogenates suggesting that studies establishing oxysterol penetration into lens fibers may be moot.

In summary, while the concept of a medical treatment for cataracts that can reverse protein aggregation to restore lens clarity is exciting, independent confirmation that oxysterol treatment, such as with lanosterol or 25-hydroxycholesterol, can restore vision is required. The results of the present studies using the established technique of whole lens culture and human lens protein binding studies, complemented with *in silico* molecular modeling and docking studies, provide no evidence that oxysterols have anti-cataractogenic activity or can bind aggregated lens protein to dissolve cataracts. We join two other laboratories in failing to independently confirm the premise that oxysterols are effective in reversing cataracts.

## Data Availability

The data that supports the findings of the reported studies are within the paper.
